# Review of Cognitive Biases in ACGME Milestones Training Assessments in Post-graduate Medical Education Programs

**DOI:** 10.7759/cureus.5518

**Published:** 2019-08-29

**Authors:** Doron Feinsilber, Duminda S Siripala, Katrina A Mears

**Affiliations:** 1 Hematology/Oncology, Medical College of Wisconsin/Froedert Cancer Center, Milwaukee, USA; 2 Nephrology, University of Pittsburgh Medical Center Altoona, Altoona, USA; 3 Ophthalmology, Retina Consultants of Southwest Florida/National Ophthalmic Research Institute, Fort Myers, USA

**Keywords:** cognitive bias, graduate medical education, reverse halo effect, psychiatry, acgme, stroop effect

## Abstract

The course of study for young physicians for post-graduate training is an exciting and life-changing opportunity, one that is filled with the relentless optimism of intellectual discovery and personal growth and development. The American Council for Graduate Medical Education (ACGME) is a non-profit private council that evaluates and accredits internship, residency, and fellowship programs. The role of the ACGME is to oversee curriculums, training environments, and specialty evaluation standards to ensure satisfactory competency leading to board eligibility and certification in the respected field of study.^ ^The ACGME has the monumental task of guiding educational standards that are designed to both protect the public welfare and further educational programs. Many educational standards are objective, such as quantitative performance on examinations, involvement in research, and involvement in systems development and quality improvement. However, key clinical performance measures are based on prior training and experience. Over the last several years, studies examining rates of abuse and discrimination during post-graduate medical training in both the United States and Canadian studies, which have reported alarmingly high rates of 50%. With the increasing utility and availability of social media, such issues have become more transparent to the public. A plethora of studies has been conducted, examining physician biases towards patients, practice changes, insurance company regulations, and evolving healthcare systems. However, a significant amount of evaluation is merited when examining individual institutional cultures and the educational environments that harbor them. We wish to examine the role of ever-evolving specialty-specific ACGME-instituted educational milestones in Internal Medicine and Opthalmology in the context of potential cognitive biases and their implementation within post-graduate training programs.

## Introduction and background

With the objective of examining the role of cognitive bias in the context of the American Council for Graduate Medical Education (ACGME)-defined specialty-specific educational milestones, a clinical definition must first be examined. The concept of cognitive bias is defined as the manner by which human beings process information and develop a hypothesis, particularly when assessing complex tasks at various stages of development. The concept of cognitive bias in educational assessments in post-graduate training programs is multi-faceted [1]. Based on regional, cultural, interpersonal, and institutional differences, a post-graduate trainee could appear well above or below expectations based strictly on perceived social reality. A significant deviation from perceived reality could create significant issues such as a false sense of grandiosity in a below-average trainee and an inability to incorporate real-time feedback. Conversely, a trainee who performs well but receives unsatisfactory ratings may develop feelings of inadequacy, anxiety, depression, and low self-esteem [2]. Either scenario could have catastrophic effects on patient care and on the entire hospital system as a whole. An examination of the hazardous personal and professional effects that institutional and departmental biases may have on trainees are reviewed. A more detailed evaluation of the role of cognitive bias in the context of current ACGME accreditation standards will be reviewed and evaluated.

## Review

The halo effect in cognitive psychiatry is defined as an individual having a desirable trait that affects perceptions more universally and broadly [3]. The definition of a desirable trait could range anywhere from physical appearance, presentation on social media, charisma, or institutional and cultural identification with a hospital and department. Examples may include being of the same denomination as a religiously affiliated hospital or department or being from the same geographic area as the faculty in a particular training program. The halo effect could potentially be hidden or omitted from individuals interviewing for programs governed by regulating bodies such as the ACGME [3]. Given the limited supply and increasing demand curve for residency and fellowship positions, both applicants and faculty may have a propensity to bypass potential warning signs of toxicity within a training program. This would allow applicants to have a greater advantage in identifying programs that are miscible with their individual educational needs. Increasing expenditures for additional training positions from the Centers for Medicare and Medicaid Services (CMS) would be a potential pathway in limiting such omissions. The halo effect risks lowering the standards required for a superior evaluation and a trainee’s educational workload. A trainee who has already established a positive rapport with a program's culturally identified faculty risks creating a subconscious perception of competency. Thus, inappropriate variability in degrees of clinical supervision could occur.

Conversely, the concept of the reverse halo effect, also known as the devil effect, may unfairly impact the evaluation of a resident or fellow by faculty. An undesirable trait in a trainee may become representative of most, if not all, milestone competencies being assessed. Based on geographic, cultural, and institutional norms, the definition of an "undesirable" trait can be variable. It's not uncommon for faculty to sublimate personal biases that create a focal point in what determines an undesirable trait. In the context of a training program where a program director has a disproportionate amount of power, this form of bias may potentially have catastrophic consequences on a young physician’s career, reputation, and livelihood. The ACGME has made strides in promoting against institutional nepotism. In comparison to early career and financially limited trainees, large hospital systems have significant legal and financial resources with which to assert their will and public image to the Centers for Medicare and Medicaid Service (CMS) and accreditation bodies such as the Joint Commission. In the hierarchy of a hospital system, a program director and section chief are much more likely to support their own faculty rather than a trainee, even with little direct evidence [3].

The reverse halo effect could also be acquired when a member in a department wishes to raise their own stature by tacitly disparaging another member of the department. Frequent assassinations of trainees' reputations within a program may lead to the development of a scapegoat culture, especially as such programs are likely to foster a herd mentality. The change in perception does not occur immediately and could develop over months or even a year. The result of such biases are feelings of inadequacy, stress, and depression in previously motivated and dedicated trainees [1]. Programs not only need to address these biases early but also implement more effective self-care programs and counseling for trainees. Inconsistencies due to an unregulated or under-acknowledged reverse-halo effect could tacitly position training programs in dangerous violation of accreditation standards [3]. To illustrate, specific core requirements taken directly from the ACGME accreditation standards will next be reviewed and followed by a hypothetical scenario demonstrating a potential violation due to bias.

ACGME program requirements are broad and encompass a vast variety of competencies on both house-staff and faculty. A core requirement, for example, is that "the faculty must evaluate fellow performance in a timely manner during each rotation or similar educational assignment and document this evaluation at the completion of the assignment." To illustrate, if a faculty member has a personality conflict with a trainee, the motivation for completing an evaluation in real-time markedly diminishes. In doing so, the educational and personal growth utility of performance evaluations decreases [4-5]. Adherence to this requirement is not so uncommon. Bias by faculty may notably not only result in inaccurate assessments but also create a critical lag time in capturing an easily correctable deficiency in real-time. It has been noted by educational psychiatrists that real-time feedback results in stronger cognitive memory and synthesis of new information.

ACGME milestones project

The Milestones Working Groups for all accredited specialties and subspecialties were initially established by a joint venture with the Accreditation Council for Graduate Medical Education (ACGME), the American ‘Board of Internal Medicine (ABIM), and the Alliance for Academic Internal Medicine (AAIM) and involved both community physicians and academicians. Individually, tailored specialty-specific milestones were constructed into several dozen metrics that each required satisfactory ratings for trainees for promotional decisions and the establishment of board eligibility. At the completion of each monthly rotation, an online assessment through GME-approved software is completed with specific ratings, each of which is tethered to a core competency. These competencies are with respect to professionalism, medical knowledge, problem-based learning, procedural skills, and interpersonal skills. Typically, each educational goal is assessed on a scoring scale from one to nine, with a five or higher being defined as a satisfactory rating. The milestones within individual specialties are evaluated throughout the course of a trainee's education to identify individual educational trajectories and deficiencies, the goal being to achieve a "ready for unsupervised practice" status on individual milestones. Each component of the evaluation process has an inherent propensity for cognitive bias from a program and faculty perspective. We wish to examine the correlations in cognitive bias with official ACGME-defined educational milestones in further detail.

Within surgical specialties such as Ophthalmology, the 360-degree longitudinal milestones include both surgical and non-surgical evaluations. Within medical specialties, such as Internal Medicine, a broader evaluation is evident, which includes the synthesis of complex tasks and preparation for serving as both a generalist and a consultant. The scope of practice, as defined with the ACGME and the American Board of Ophthalmology (ABO), for example, requires trainees to achieve competency in the management of common toxicities associated with the treatment of central nervous system (CNS) lymphoma, endophthalmitis, retinal detachment and common identifications of optic neuropathy [6-8]. With the increasing complexity and breadth of medical knowledge and synthesis, it is imperative that faculty in training programs entrusted with the welfare of their learners not only be up to date through Maintenance of Certification (MOC) but also through peer-reviewed research and targeted specialty-specific continuing medical education (CME).

Evaluation of cognitive bias in ACGME milestones 

To illustrate the role of cognitive bias in post-graduate training, in-depth comprehension of metrics being evaluated must be established. The ACGME publishes specialty and subspeciality-specific metrics and training programs must evaluate trainees based on them. A notable example is in Medical Oncology, which is constantly evolving with respect to the understanding of novel targeted therapies and grade three and four toxicities associated with immune checkpoint inhibitors utilized in the management of both solid tumors and hematologic malignancies [9]. The ACGME milestones project ensures that trainees in Medical Oncology are aware of common adverse effects and contraindications to standard cytoreductive chemotherapy regimens such as with high dose methotrexate [8]. Training programs must recognize cross-specialty learning objectives, such as in palliative care, in order to create a complete and longitudinal educational experience. Within the ACGME milestones in Medical Oncology, programs exist to evaluate fellows on procedural and technical expertise in bone marrow biopsies and intrathecal chemotherapy in addition to the management of central venous catheters utilized in the administration of chemotherapeutic agents and targeted therapies. During a fellowship program, faculty could have variable degrees of expectations in the technique of bone marrow biopsies and aspirations. For example, a fellow may be technically proficient, however, he may not be able to obtain an appropriate core sample due to a patient's clinical status, which is oftentimes found in conditions such as myelofibrosis or "dry tap." If an Oncology fellow is unable to obtain an appropriate sample, a faculty could easily label this as a technical error and fail the trainee on this particular educational milestone. Here, we see an integration of metrics established by the ACGME milestones and the role of cognitive bias in the evaluation process. Studies have examined the milestones' impact on evaluations with narrative comments and the perception of feedback for internal medicine residents and found residents did not feel the milestones project improved these [10]. There can be a significant delay between rotation completion and timely faculty feedback, which erodes the educational utility of feedback [11].

An additional example to illustrate disparities in cognitive bias and ACGME-defined clinical milestones used for the purposes of evaluation and promotion is within the subspecialty of Nephrology. It is imperative that trainees achieve competency in the diagnosis and management of acute kidney injury, including prerenal etiologies, such as volume depletion, intrinsic causes such as the myriad types of glomerulonephritis and tubulointerstitial injury, and postrenal causes such as obstructive uropathy. In addition, fellows must achieve sufficient training and experience with regard to the management of chronic kidney disease and its attendant complications and should develop confidence with regard to both conservative management and renal replacement therapy in patients with more advanced chronic kidney disease. Experience and training with regard to renal replacement therapies should encompass conventional hemodialysis, continuous renal replacement therapy, and peritoneal dialysis. The faculty in nephrology fellowship programs must also provide sufficient guidance and constructive feedback when training fellows to perform common nephrology procedures, including renal biopsy and temporary dialysis catheter placement. Nephrology faculty still must master the art of instilling confidence in their trainees when performing these procedures while, at the same time, always perform due diligence in ensuring patient safety. The same holds true with regard to the diagnosis and management of both acute and chronic kidney disease, in both inpatient and outpatient settings. The examples of knowledge and skills listed above, which nephrology trainees must master provide a framework upon which milestones may be tailored for renal fellowship programs. For faculty in Nephrology fellowships, the idea of using the milestones as objective criteria for the determination of promotion or remediation of fellows is still in its infancy. In addition, feedback offered must be both constructive and specific. Feedback that is vague and provides no suggestions for improvement is counterproductive to the growth of knowledge and skills in trainees.

Although the ACGME has defined clinical learning milestones in Ophthalmology residency programs, the subspecialty fellowship training positions currently have no governance by the ACGME; instead, the Association of University Professors of Ophthalmology (AUPO) is the governing body. The AUPO FCC was established for the maintenance of standards for subspecialty fellowship training in ophthalmology. While having a governing body is absolutely necessary, unfortunately, the reporting of compliance by programs with the requirements for AUPO is based on self-reporting evaluations by the program director at that program, thereby introducing the first layer of bias in an evaluation system whose aim is ultimately to give objectivity to the compliance process. With less oversight, a program director could risk a higher chance of non-compliance with program requirements. As such, a program could present future employers, insurance companies, and patients with an inaccurate impression of the professional stature of a program.

The AUPO has a comprehensive list of requirements for program directors that states that program directors engage in ongoing research in the area of retina and vitreous as demonstrated by publishing in peer-reviewed journals, presentations of research material at national meetings, or both. Self-reporting, gone unregulated in many cases, maybe erroneous unless verified individually through primary source verifications. Program directors must ensure, direct, and document the adequate supervision of fellows at all times. Fellows must be provided with rapid, reliable systems for communicating with supervising faculty. In some programs where a program director has an adjunct or partial academic appointment, it is difficult to quantify how that program can be in compliance with accreditation and quality metrics.

To illustrate, an eye examination room with a slit lamp should be easily accessible in the hospital; however, this is often not available in poorly equipped hospitals and poorly funded hospitals. There must be access to current diagnostic equipment, including ultrasonography and fluorescein angiography. In certain clinics, this equipment is outdated, even nonfunctional, and in some cases, an outside contractor with little or of questionable credentials is scheduled to periodically perform these tests when the academic center is not adequately funded or has poor departmental leadership unable to secure funds to buy basic equipment. This would apply particularly to the provision of fluorescein angiography and modern ultrasonography equipment, equipment essential to the functionality of any vitreoretinal fellowship.

With respect to the broad ACGME-defined milestone of scholarly activity, programs are supposed to track and evaluate a resident and fellows' progress with respect to quality improvement, clinical and translational research, and didactic activity involvement. The scholarly activity milestone by the ACGME is a mandatory one that trainees participate in a minimum of 100 hours of didactic instruction, including seminars, lectures, and approved basic science courses, of which at least 50 hours will be in the parent institution. All too frequently, these requirements are not met due to a lack of oversight by the program director, poorly motivated fellows with inadequate supervision, and extensive distances between clinical sites whereby a functional program is not attainable. The standards also require that a fellow should attend local and regional conferences relevant to vitreoretinal diseases and that research activities are required of all retina and vitreous fellows; however, in lower-tier programs who claim to have these credentials, there is no direct verification of the credibility of this information. Even though the program must meet or exceed RRC requirements for the teaching of ethics in residency and fellowship programs, this is difficult to comply with in situations where a program director spends the bulk of time in independent private practice. Institutional biases have been shown to take a psychological toll and contribute to physician burnout [12]. Recent papers have revealed these biases may vary by specialty, largely dependent on the gender's representation in that specialty and disproportionately affect women in specialties that have a disproportionately high male to female ratio [13]. There have been several efforts to integrate the presence of implicit bias into the education of health professionals. One recent study even proposed a six-point actionable framework for integrating implicit bias recognition and management into health professions' education [14]. Other approaches to deal with bias have included an observed structured teaching encounter [15-16].

Scholarly activity and quality improvement projects as a key educational milestone

The concept of quality improvement is a valuable and essential educational tool in training programs and general medical practice. The emphasis to engage in quality improvement projects has become a focal point embedded within the milestones project. These projects encourage introspection, team building, and humility within a department at all levels of training and expertise. By accomplishing the task of systems improvement respect through quality improvement projects, a culture of safety and enhanced communication is realized. Secondary outcomes that integrate both patient safety and the maximization of institutional resources ultimately creates better patient outcomes and enhances the synthesis of complex clinical concepts from an educational perspective. For the purposes of institutional and educational improvement, the Institutional Review Board (IRB) is an incredibly valuable resource in supporting such endeavors as retrospective studies. An example is a large academic cancer center being mindful of the appropriate use of intravenous immunoglobulin (IVIg) in patients with hypogammaglobulinemia [17]. Scholarly activity, such as quality improvement projects, are an integral part of any training program, and institutional cognitive biases may affect the ultimate outcome of such endeavors that ultimately affect patient outcomes and educational advancement [18-20]. Residents' and fellows' performance on milestones will become a source of specialty-specific normative data for use by the Specialty Review Committees in the assessment of the quality of residency and fellowship programs and the facilitation of improvements to program curriculum, thereby allowing improvements in trainee performance.

Need for uniform standards, including remediation, probation, and termination, in post-graduate training programs

The process for hospital graduate medical education (GME) committees in formal assessments and promotional decisions allows faculty to ensure the trainee is achieving requisite educational advancements. Trainees perceived to be achieving the specialty or sub-specialty milestones are considered in "good standing." However, due to a lack of standard definitions of satisfactory standing, trainees who have been subjects of cognitive bias may become easy targets for retaliation by faculty [5]. A common phenomenon is that trainees will achieve different milestones at varying times throughout the course of a training program. These milestones may include the development of emotional maturity, professionalism, and enhancements in medical knowledge. Not uncommonly, a trainee will undergo a period of informal remediation if a milestone has not been yet achieved. A formal period of remediation would involve GME and would be noted in a trainee's file. However, it would not be shared during the application for medical licensure and medical staff privileges. From an institutional perspective, a program director wishing to create a safe administrative pathway from a legal perspective toward the removal of a resident or fellow would then convert a formal remediation into probation. Initiation of probation would include documentation extending into the final summative evaluations and employment verifications. The transition between remediation and administrative probation has a much higher degree of variability and subjectivity.

A significant ACGME core program requirement is, "Be responsible for monitoring fellow stress, including mental or emotional conditions inhibiting performance or learning, and drug- or alcohol-related dysfunction." A program director and leadership have a moral and ethical responsibility to ensure that the training environment and the culture of a department and sponsoring institution are conducive to learning [5]. Institutions have a significant amount of resources to omit the evidence of non-compliance to third parties, making abuse even more difficult to prove. A program director with cognitive bias will utilize mostly negative outcomes without a root-cause analysis in defending promotional and disciplinary actions toward a trainee.

It is not uncommon for a diligent trainee to be selectively blamed for negative repercussions resulting from institutional disorganization, such as an overbooked clinic, for example. Due to a corrosive environment and institutional disorganization, the program director may decide to believe negatives that are otherwise entirely divorced from reality. Learning in such a hostile environment is often exceedingly difficult for a trainee. Scenarios such as these violate the ACGME core milestones requirements to "administer and maintain an educational environment conducive to educating trainees in each of the ACGME competency areas" and "the physician faculty must meet professional standards of ethical behavior" [5].

The Stroop effect was first described in the field of cognitive psychiatry by Dr. John Ridley Stroop in 1935, when studies were found to evaluate interference in the reaction times of a task. The working theory behind the Stroop effect is the mind quickly interprets the semantic meaning over the actual meaning. As such, a faculty member with a strong positive or negative predisposition may have difficulty in deciphering information on a milestone due to extraneous tangential information. The Stroop effect can be characterized as either "interference," in which one cognitive operation degrades the perception of another, or "facilitation," in which a cognitive operation can enhance the perception of the same task [4].

A vast number of prior studies have displayed a propensity for implicit bias against those trainees with disabilities. For example, disabilities such as Asperger's syndrome, hearing impairment, a mobility issue, or a speech impediment can often be magnified into an “incompetency” and affect evaluations if a supervising attending has a particularly strong negative cognitive bias towards those with disabilities. This highlights the importance of raising awareness, educating, and even performing standardized testing routinely among supervising faculty about such biases, thus mitigating against the effects of cognitive bias as much as possible [2].

Institutions sponsoring post-graduate training programs have an arduous task with respect to creating fair and standardized evaluation and promotion standards. During training, learners come from diverse cultural, emotional, and psychological backgrounds, which could subconsciously affect an evaluator's judgment. By acknowledging both the institutional and cognitive limitations in creating more objective evaluation standards consistent with the ACGME-sponsored milestones, a more inclusive and fertile ground for education and mentorship could be established.

## Conclusions

It is apparent that sponsoring institutions may require additional safeguards from the risk of institutional nepotism. There are various strategies to accomplish this, including the neuropsychological testing of faculty, assigning each trainee an academic advisor who is faculty at a separate ACGME-accredited program, and Cognitive Bias Modification Therapy (CBMT) for faculty.
